# Co-Resistance Structure and Multidrug Resistance-Associated Antimicrobials in *Escherichia coli* from Healthy Pigs in Japan: A Computational Analysis of JVARM Data, 2012–2023

**DOI:** 10.3390/antibiotics15050441

**Published:** 2026-04-29

**Authors:** Yuta Hosoi, Michiko Kawanishi, Mari Matsuda, Saki Harada, Maika Kubo, Hideto Sekiguchi

**Affiliations:** Veterinary AMR Center, National Veterinary Assay Laboratory, Ministry of Agriculture, Forestry and Fisheries, Ibaraki 205-8535, Japan

**Keywords:** AMR, co-resistance, multidrug resistance, XGBoost, SHAP, JVARM, XAI

## Abstract

**Background/Objectives:** The Japanese Veterinary Antimicrobial Resistance Monitoring System (JVARM) conducts longitudinal monitoring of antimicrobial resistance (AMR) in indicator bacteria from food-producing animals. For *Escherichia coli* from healthy pigs, slaughterhouse-based sampling has been conducted for approximately a decade, yielding a substantial accumulation of MIC data. While JVARM reporting has traditionally focused on annual resistance proportions by drug, the availability of long-term data enables investigation of cross-drug relationships, including MIC similarity and co-resistance patterns. This study aimed to (i) identify the co-resistance structure among antimicrobial agents using MIC- and phenotype-based similarity measures and (ii) identify drug resistances most strongly associated with multidrug resistance (MDR). **Methods:** We analyzed broth microdilution MIC data obtained annually for *E. coli* isolates from healthy pigs in the JVARM program in Japan between 2012 and 2023. Antimicrobial resistance was classified from MIC results and annual resistance prevalence was calculated for each antimicrobial. For the co-resistance and MDR analyses, isolate-level data were pooled across the full study period. To identify co-resistance structure, we performed hierarchical clustering using (i) correlation-based similarity of MIC profiles and (ii) Jaccard similarity of binary resistance profiles (resistant/susceptible classification). Multidrug resistance (MDR; ≥3 antimicrobial classes) was further modeled using XGBoost with each drug resistance as a predictive feature, and feature contributions were evaluated using gain, permutation importance, and SHAP values. We also examined how SHAP-based attributions varied when the outcome definition was set to ≥1-, ≥2-, or ≥3-class resistance. **Results:** Within the study period, resistance remained highest for tetracycline and moderate for streptomycin, ampicillin, sulfamethoxazole–trimethoprim, and chloramphenicol, whereas resistance to other agents was low. MIC-based correlation analysis revealed coordinated variation among ampicillin, sulfamethoxazole–trimethoprim, streptomycin, chloramphenicol, and tetracycline. Separately, Jaccard similarity of binary resistance profiles identified two closely positioned co-resistance groupings (Ampicillin/Streptomycin/Tetracycline and chloramphenicol/sulfamethoxazole–trimethoprim). Ampicillin was identified as the medoid in both MIC-based and resistance-profile similarity spaces, with streptomycin also positioned near the center in both structures. In the XGBoost model for MDR (≥3 classes), ampicillin resistance was consistently the highest-contributing feature when evaluated by gain, permutation importance, and SHAP. When we examined how SHAP-based attributions varied across outcome definitions (≥1-, ≥2-, and ≥3-class resistance), feature importance largely followed resistance prevalence at ≥1–≥2 classes (tetracycline highest) but shifted at ≥3 classes to ampicillin as the top feature. **Conclusions:** Both MIC-based and phenotype-based analyses revealed co-resistance structures. Under the MDR definition used in this study, explainable machine-learning analyses showed that ampicillin resistance emerged as a leading resistance feature associated with MDR. Because these findings are associative rather than causal, further work will be needed to clarify mechanisms. These findings have important implications for antimicrobial resistance control in the Japanese pig sector, indicating that stewardship strategies may need to be tailored according to antimicrobial class and underlying co-resistance structure.

## 1. Introduction

Antimicrobial resistance (AMR) has been widely recognized as a major global public health challenge and is often described as a “silent pandemic” that gradually undermines the effectiveness of infectious disease treatment [1]. AMR is not confined to a single sector; rather, it is an interconnected issue spanning human, animal, and environmental domains. Consequently, coordinated actions under the One Health framework are required to achieve sustainable reductions in AMR across these sectors [2].

In veterinary and livestock production systems, antimicrobials are essential medical resources that support animal welfare, productivity, and food safety. However, antimicrobial use (AMU) can exert selective pressure that promotes the selection and persistence of resistant bacteria and resistance genes. Therefore, balancing prudent use (stewardship) with continuous surveillance is critical [3]. In Japan, the Ministry of Agriculture, Forestry and Fisheries has conducted systematic surveillance of AMR trends in animal-derived bacteria and AMU through the Japanese Veterinary Antimicrobial Resistance Monitoring System (JVARM) since 1999 [4].

Long-term surveillance data from JVARM indicate that reductions in antimicrobial use do not always result in proportional decreases in resistance prevalence. For example, resistance to third-generation cephalosporins in *Escherichia coli* from healthy chickens declined following reductions in off-label in ovo use during the 2010s [4]. In contrast, in the Japanese pig sector, overall veterinary antimicrobial use—particularly tetracyclines—has shown a declining trend since the late 2010s, whereas tetracycline resistance in *E. coli* from healthy pigs has remained persistently high despite reduced AMU [4].

At a regional scale, cross-national analyses conducted in the European Union have generally reported positive associations between AMU and AMR across countries, although the strength of these relationships varies substantially depending on antimicrobial class and bacterial species [5]. At the national level, several studies have concluded that reductions in AMU are associated with decreases in AMR [6,7,8]. However, a study from the Netherlands reported that although antimicrobial use in livestock was substantially reduced, declines in resistance were primarily observed for newer or less frequently used agents—such as third- and fourth-generation cephalosporins and fluoroquinolones—whereas resistance to historically widely used antimicrobials, including penicillin and tetracyclines, was less responsive to reductions in use [9]. At the farm level, longitudinal studies have reported that resistance persistence is not primarily determined by AMU alone, but instead reflects complex interactions involving historical antimicrobial use, co-selection, multidrug-resistant plasmids, and environmental reservoirs of resistant bacteria [10].

These observations, together with the pattern observed for tetracycline in JVARM, motivated us to examine patterns of co-resistance among antimicrobial agents in *E. coli* from healthy pigs in Japan and to assess whether underlying co-resistance structure could help explain why reductions in AMU do not always lead to proportional declines in resistance prevalence.

In AMR surveillance of food-producing animals, the intestinal commensal bacterium *E. coli* is widely used as an indicator organism. Because *E. coli* is present across human, animal, and environmental sectors, it provides a common reference point for comparisons across different reservoirs within the One Health framework [11,12]. Accordingly, this study focused on *E. coli* isolates obtained from rectal fecal samples of healthy pigs collected at slaughterhouses.

Machine-learning methods have increasingly been applied across a wide range of scientific fields, and AMR research is no exception [10]. Broadly, data-driven approaches in AMR analysis can be divided into unsupervised methods for exploring latent structure in resistance data [13,14] and supervised methods for modeling predefined resistance outcomes, particularly for predicting resistance phenotypes from other available features [15,16,17].

In this study, co-resistance structure among antimicrobials was first examined using unsupervised, similarity-based analyses. Pairwise relationships between drugs were quantified using Pearson correlations of MIC profiles and Jaccard similarities of binary resistance profiles, and these relationships were summarized by hierarchical clustering. Because Jaccard similarity can be influenced by the marginal prevalence of resistance for individual drugs, observed pairwise Jaccard values were additionally compared with Monte Carlo-derived null expectations under an independence model to assess whether the observed co-occurrence exceeded that expected from marginal resistance frequencies alone.

To complement the analysis of co-resistance structure, we next examined which antimicrobial resistances were most strongly associated with multidrug resistance (MDR) using supervised multivariable modeling. Within the broader framework of multivariable analysis, classical regression-based models have long been used to assess associations between explanatory variables and outcomes and remain useful because of their interpretability. However, regression models may have limited ability to represent complex nonlinear relationships and higher-order interactions. In contrast, many machine-learning models offer greater flexibility for capturing such complexity, although they often do so by increasing internal model complexity [18]. For example, XGBoost has been widely used and often performs strongly on tabular data [19]. It generates predictions by sequentially adding decision trees, and each new tree focuses on correcting the errors made by the previous trees [20]. In general, increased modeling flexibility in many machine-learning methods is often accompanied by reduced direct interpretability. To address this limitation, explainable artificial intelligence (XAI) methods have been developed to support post hoc interpretation of complex models [21]. In particular, SHAP (Shapley Additive Explanations) enables the decomposition of model predictions into feature-level contributions and has been widely used to interpret complex predictive models [22]. In recent years, the combination of machine-learning models and XAI techniques, including XGBoost interpreted with SHAP, has also been applied in AMR research [17].

In the present study, MDR, defined as resistance to three or more antimicrobial classes following a commonly used definition [23], was modeled using XGBoost with drug-level binary resistance indicators as explanatory variables. Because the outcome is defined directly by combinations of the explanatory variables themselves, the purpose of this analysis was not to optimize prediction but to identify which drug resistances contribute most strongly to MDR classification. To interpret the fitted model, we applied XAI methods and complementary feature-importance measures, including SHAP, permutation feature importance, and gain-based feature importance.

By integrating unsupervised analyses of co-resistance structure with supervised modeling of MDR, this study characterizes co-resistance patterns and identifies the antimicrobial resistances most strongly associated with MDR in *E. coli* from healthy pigs in Japan. These analyses move beyond conventional reporting of drug-specific resistance proportions and provide findings with potential relevance to antimicrobial stewardship.

## 2. Results

### 2.1. Trends in Resistance Prevalence

**Figure 1:**
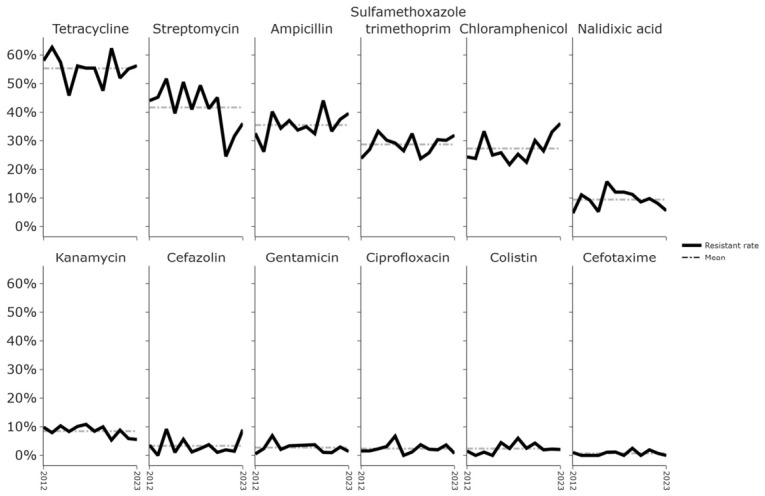
Trends in Antimicrobial Resistance of *Escherichia coli* Isolates from Pigs, 2012–2023.

The annual resistance prevalence from 2012 to 2023 is shown in Figure 1. Over the 12-year period, the mean resistance prevalence was highest for tetracycline, remaining consistently elevated at 55%. Moderate levels of resistance were observed for streptomycin (42%), ampicillin (36%), sulfamethoxazole–trimethoprim (29%), and chloramphenicol (27%). Resistance prevalence for all other agents was ≤10%, including nalidixic acid (9%), kanamycin (8%), cefazolin (3%), gentamicin (3%), ciprofloxacin (2%), colistin (2%), and cefotaxime (1%).

Notably, resistance to antimicrobials of clinical importance in human medicine—including the third-generation cephalosporin cefotaxime, the fluoroquinolone ciprofloxacin, and colistin—remained low throughout the study period.

Temporal trends in resistance prevalence were evaluated using the Mann–Kendall trend test. At the unadjusted significance level of 0.1, chloramphenicol and streptomycin exhibited trends suggestive of monotonic change. Sen’s slope corresponded to an increase of 0.6 percentage points per year for chloramphenicol and a decrease of 1.1 percentage points per year for streptomycin. However, no trend remained significant after Benjamini–Hochberg adjustment at the 0.1 level, and no statistically significant trend was observed at the 0.05 level either before or after adjustment. Because this analysis was exploratory and intended to identify broad temporal patterns rather than to formally test specific hypotheses, these findings were interpreted as indicative of general tendencies rather than conclusive evidence.

### 2.2. Clustering and Correlation Structure of MIC Profiles

**Figure 2:**
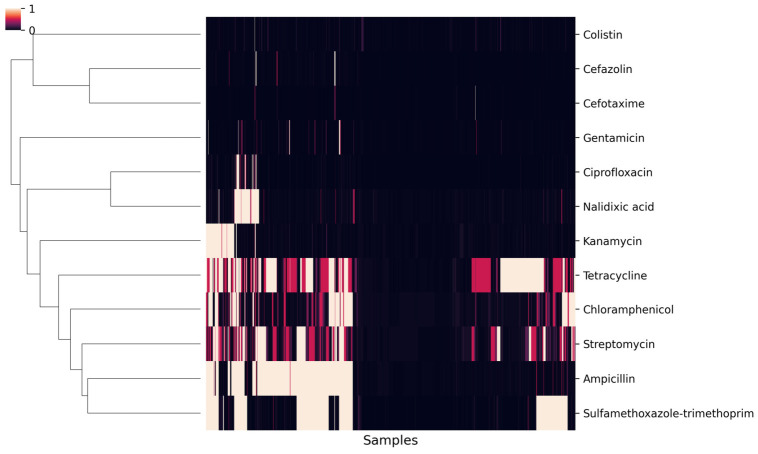
Correlation-Based Clustered Heatmap of MIC Values for Antimicrobial Agents in *E. coli* Isolates from Pigs, 2012–2023.

Hierarchical clustering of normalized MIC profiles (Figure 2) showed distinct clusters among antimicrobial agents. As described in the Methods, drug–drug similarity was computed using Pearson correlation coefficients between normalized MIC profiles, and hierarchical clustering was performed using a correlation-based distance.

Quinolones, represented by ciprofloxacin and nalidixic acid, formed a coherent cluster, supported by a correlation coefficient (r) of 0.55. Within the β-lactam category, the cephalosporin pair cefazolin and cefotaxime also formed a coherent cluster, supported by a correlation coefficient (r) of 0.44.

Beyond these major class-based clusters, several cross-class associations were also observed. Ampicillin was associated with multiple cross-class correlations, including with sulfamethoxazole–trimethoprim (r = 0.43), streptomycin (r = 0.43), chloramphenicol (r = 0.38), and tetracycline (r = 0.32). Sulfamethoxazole–trimethoprim also correlated with chloramphenicol (r = 0.39) and streptomycin (r = 0.37).

Additional modest correlations included chloramphenicol with tetracycline (r = 0.29), cefotaxime with colistin (r = 0.28), streptomycin with tetracycline (r = 0.27), chloramphenicol with streptomycin (r = 0.25), sulfamethoxazole–trimethoprim with tetracycline (r = 0.25), chloramphenicol with kanamycin (r = 0.24), kanamycin with tetracycline (r = 0.21), and chloramphenicol with ciprofloxacin (r = 0.21).

In contrast, most drug pairs (76%, 50/66) showed weak or negligible correlations (r < 0.20), suggesting that MIC co-variation was limited overall and that most unrelated drug pairs followed largely independent patterns.

Using the distance-sum metric (Σ(1 − r)), ampicillin had the smallest total distance to all other agents (8.56) and was therefore identified as the medoid. Streptomycin (8.89), chloramphenicol (8.94), and sulfamethoxazole–trimethoprim (9.02) were the next closest agents in correlation space.

### 2.3. Clustering and Jaccard Similarity Structure of Resistance Profiles

**Figure 3:**
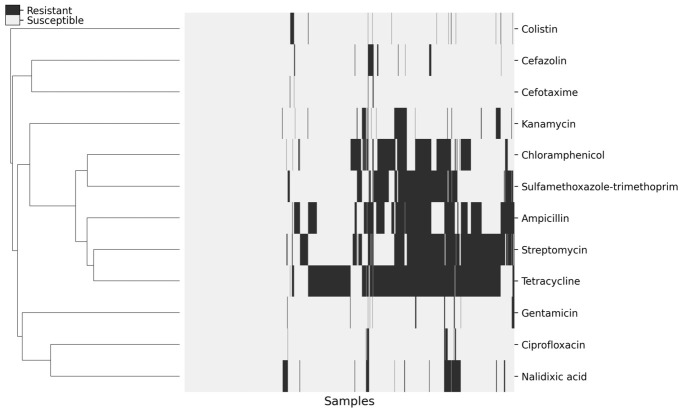
Clustered Heatmap of Resistance patterns for Antimicrobial Agents in *E. coli* Isolates from Pigs, 2012–2023.

Hierarchical clustering using Jaccard similarity of binary resistance profiles revealed distinct co-occurrence patterns among drugs (Figure 3). Two closely connected clusters were evident. The first cluster was centered on ampicillin, streptomycin, and tetracycline, with the strongest overlap observed for streptomycin with tetracycline (J = 0.51), followed by ampicillin with streptomycin (J = 0.47) and ampicillin with tetracycline (J = 0.46). The second cluster was characterized primarily by chloramphenicol and sulfamethoxazole–trimethoprim (J = 0.47). These clusters were closely linked, however, by high similarity between streptomycin and sulfamethoxazole–trimethoprim (J = 0.45), as well as by ampicillin with sulfamethoxazole–trimethoprim (J = 0.43) and ampicillin with chloramphenicol (J = 0.41), placing the two clusters adjacent in the dendrogram.

In contrast, most remaining pairs showed low Jaccard similarity. The aminoglycoside pair gentamicin–kanamycin displayed minimal overlap (J = 0.01). Colistin showed uniformly low similarity across all comparisons (range: J = 0.01–0.05). Cefotaxime likewise showed very low overlap with most drugs (range excluding the cefazolin pair: J = 0.01–0.05), despite a modest within-class similarity with cefazolin (J = 0.15). Ciprofloxacin showed limited cross-class overlap (J = 0.04–0.11), peaking with gentamicin (J = 0.11) and cefazolin (J = 0.07), while showing higher within-class similarity to nalidixic acid (J = 0.26).

Using the distance-sum metric (sum of Jaccard distances to all other drugs), ampicillin was identified as the medoid (minimum total distance; sum = 8.71). Streptomycin (8.78) and sulfamethoxazole–trimethoprim (8.81) were the next closest agents, followed by tetracycline (8.86) and chloramphenicol (8.94). In contrast, cefotaxime (10.66) and colistin (10.71) were among the most peripheral agents.

**Figure 4:**
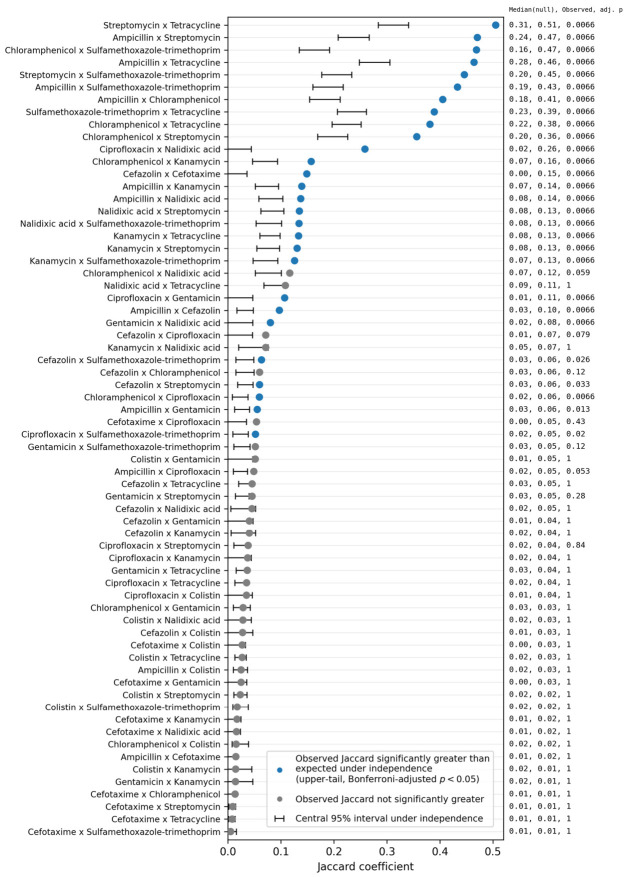
Comparison of observed and Monte Carlo-derived null distributions of pairwise Jaccard similarities.

To examine whether the co-occurrence patterns revealed by the Jaccard-based clustering could be explained solely by the overall resistance frequencies of each antimicrobial considered individually, we compared the observed pairwise Jaccard similarities with Monte Carlo-derived null expectations generated under an independence model (Figure 4). Observed similarities among drug pairs forming the major high-similarity group—particularly combinations involving ampicillin, streptomycin, tetracycline, chloramphenicol, and sulfamethoxazole–trimethoprim—consistently exceeded the corresponding null expectations after correction for multiple testing. This indicates that the strong overlaps within this group were not driven simply by chance co-occurrence resulting from individual resistance prevalences.

### 2.4. Model-Based Feature Importance Under the MDR Definition Adopted in This Study

The annual prevalence of multidrug resistance (MDR; resistance to ≥3 antimicrobial classes) fluctuated over the study period rather than showing a clear monotonic increase or decrease. Across all years combined, 523/1312 isolates (40%) met the MDR definition. Year-specific prevalence ranged from 32% (2021) to 51% (2014), with annual values of 35% (2012), 37% (2013), 51% (2014), 41% (2015), 45% (2016), 39% (2017), 41% (2018), 38% (2019), 43% (2020), 32% (2021), 43% (2022), and 40% (2023).

**Figure 5:**
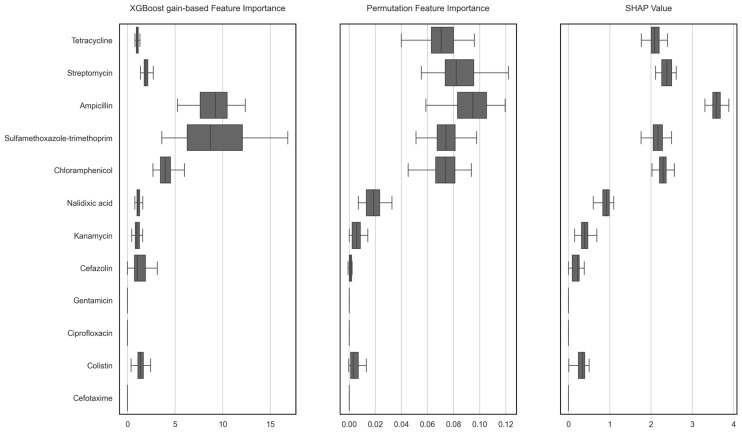
Feature importance for MDR prediction across repeated cross-validation.

To identify antimicrobial-resistance features most strongly associated with MDR classification, we trained an XGBoost classifier using MDR status as the outcome variable and drug-level binary resistance indicators as explanatory variables and evaluated it using repeated stratified cross-validation (5 folds × 10 repeats; 50 fitted models). Feature importance was assessed using three complementary approaches—XGBoost gain, permutation feature importance (PFI), and SHAP-based importance—and summarized across folds using the median (Figure 5).

Feature importance was summarized across folds using the median. By gain, ampicillin and sulfamethoxazole–trimethoprim ranked first and second, respectively (9.23 and 8.70), followed by chloramphenicol (3.96). By PFI, ampicillin showed the largest decrease in balanced accuracy (0.095), followed by streptomycin (0.082) and sulfamethoxazole–trimethoprim (0.074). By SHAP-based importance, ampicillin again ranked highest (3.58), followed by streptomycin (2.38) and chloramphenicol (2.29).

For completeness, we also report mean balanced accuracy, which was 0.99 (SD 0.01; range 0.97–1.0) across the 50 test folds. This high score was expected because MDR status was defined by a combination of drug-resistance variables used as predictors.

### 2.5. Changes in Model-Based Feature Importance Across Outcome Definitions (≥1, ≥2, and ≥3 Resistant Classes)

**Figure 6:**
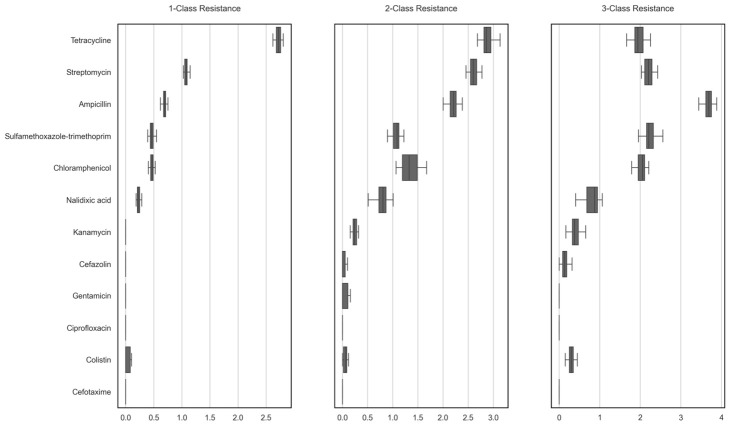
Drug-level feature importance differs across 1-class, 2-class, and 3-class resistance outcomes.

To complement the MDR feature-importance analysis (Figure 5), where MDR was defined as resistance to ≥3 antimicrobial classes, we examined how drug-level feature attributions varies across outcome definitions (≥1-class, ≥2-class, and ≥3-class resistance) using XGBoost (Figure 6).

The set and ranking of the most influential drug features differed by outcome definition. For ≥1-class resistance, the highest SHAP-based importance values (median across folds of mean absolute SHAP values) were observed for tetracycline (2.81), followed by streptomycin (1.16) and ampicillin (0.71). For ≥2-class resistance, tetracycline (2.91), streptomycin (2.69), and ampicillin (2.35) remained the top-ranked predictors. In contrast, for ≥3-class resistance, ampicillin was the highest-ranked feature (3.58), followed by streptomycin (2.38) and chloramphenicol (2.29). Tetracycline, which ranked first for ≥1- and ≥2-class resistance, was not among the top three features for ≥3-class resistance. Balanced accuracy was near perfect across outcome definitions (mean: 1.00 for ≥1-class, 0.99 for ≥2-class, and 0.99 for ≥3-class resistance), as expected from how MDR was defined.

## 3. Discussion

This study leveraged 12 years of MIC data from *E. coli* isolated from healthy pigs in Japan to move beyond the traditional year-by-year reporting of resistance proportions and instead characterize (i) how resistance traits are organized across antimicrobials (co-variation of MICs and co-occurrence of resistance) and (ii) how resistance to individual drugs is associated with multidrug resistance (MDR; ≥3 antimicrobial classes).

### 3.1. Co-Resistance Structure

A key result of our analyses was that ampicillin repeatedly appeared as the central antimicrobial agent in the resistance structure: it was the medoid—defined as the agent with the smallest total distance to all other agents—of both the MIC-correlation distance matrix and the Jaccard-distance matrix derived from binary resistance profiles. In the MIC-based space, the agents next closest to this medoid were streptomycin and chloramphenicol. In the resistance-pattern (Jaccard) space, the ranking shifted slightly, with streptomycin and sulfamethoxazole–trimethoprim being the agents next closest to the medoid. The Jaccard clustering highlighted a structured pattern of resistance co-occurrence, comprising two closely positioned groupings—an ABPC/SM/TC (ampicillin, streptomycin, and tetracycline) group and a CP/ST (chloramphenicol and sulfamethoxazole–trimethoprim) group—as well as intermediate agents such as ciprofloxacin with modest cross-class overlap, and clearly peripheral agents, notably colistin and cefotaxime, with minimal co-occurrence with other drugs.

### 3.2. Implications for Resistance Reduction

Antimicrobials that tend to form co-resistance clusters may reasonably be categorized as agents belonging to the early-introduced antimicrobial classes, whereas those exhibiting more independent resistance patterns may be viewed as members of more recently introduced antimicrobial classes, albeit not universally, as exemplified by colistin [24]. This interpretation aligns with previous observations: (i) in JVARM, resistance to third-generation cephalosporins in poultry *E. coli* declined rapidly once usage was reduced [4], and (ii) in the pig sector, despite a sustained decrease in tetracycline use since the late 2010s, tetracycline resistance in *E. coli* from healthy pigs has remained high without a proportional decline [25]. Similar trends have been reported in the Netherlands, where reductions in antimicrobial use led to pronounced decreases in resistance primarily for newer or less frequently used agents (e.g., third- and fourth-generation cephalosporins, fluoroquinolones), while resistance to historically common drugs (e.g., penicillin, tetracyclines) showed limited responsiveness [9].

Our study suggests that reductions in resistance may be more readily achievable for antimicrobial agents whose resistance patterns are relatively independent of those of other drugs—such as third-generation cephalosporins (e.g., cefotaxime), quinolones (e.g., ciprofloxacin and nalidixic acid), and colistin—because resistance to these agents showed limited co-occurrence with other antimicrobials. Although MIC-based analysis identified some cross-class correlations (e.g., between cefotaxime and colistin), these associations were not reflected in binary resistance patterns defined by clinical breakpoints. In contrast, for antimicrobials involved in co-resistance modules—such as the ABPC/SM/TC and CP/ST groupings—reducing the use of a single agent alone is unlikely to yield substantial declines in resistance; instead, coordinated reductions across interconnected classes within these modules may be required.

### 3.3. Implications for Clinical Interpretation

**Figure 7:**
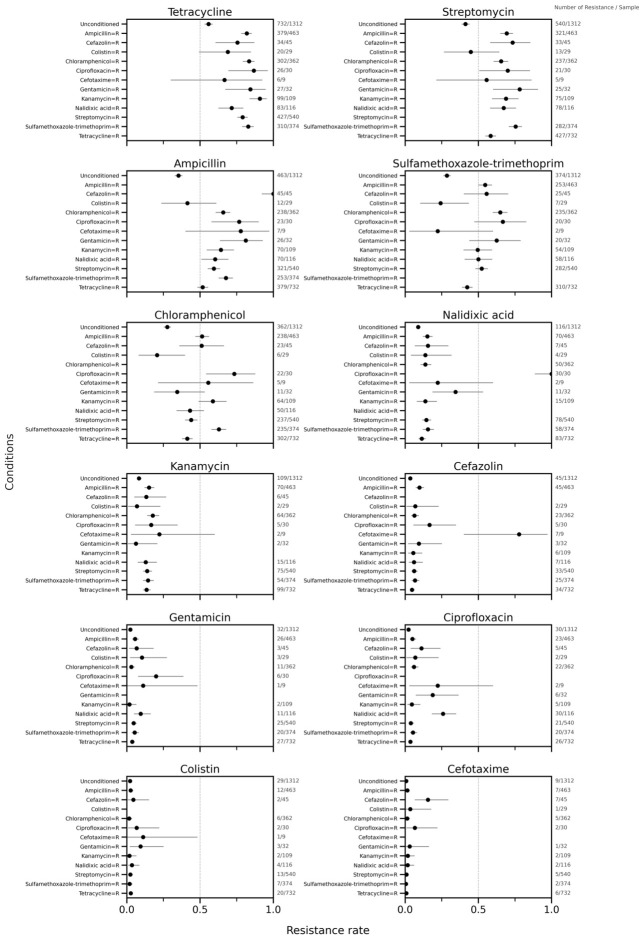
Conditional resistance rates of *Escherichia coli* isolates from pigs, 2012–2023, showing the probability of resistance to one antimicrobial given resistance to another (Resistance Escalation Antibiogram). Error bars represent the 95% confidence intervals calculated using the Clopper–Pearson exact method.

If the co-resistance patterns observed in this surveillance study are interpreted in a clinical context, resistance to one drug within a co-resistance cluster would likely be accompanied by resistance to other cluster members. Under such circumstances, where resistance is suspected or inferred—for example, from herd history, rapid testing, or empirical treatment failure—switching to another drug within the same cluster may provide limited additional benefit. For example, if ampicillin resistance is suspected or confirmed, clinicians should recognize the high probability of concurrent resistance to streptomycin, tetracycline, chloramphenicol, and sulfamethoxazole–trimethoprim. As shown in Figure 7, the conditional resistance analysis, presented here as a resistance-oriented adaptation of the escalation antibiogram concept [26], provides a more clinically interpretable view of this co-resistance structure. In this analysis, ampicillin, streptomycin, tetracycline, chloramphenicol, and sulfamethoxazole–trimethoprim showed consistently high conditional resistance rates when resistance to another agent was used as the conditioning event, whereas for most of the remaining agents, conditional resistance rates were less markedly affected by resistance to other drugs. These findings are consistent with the core co-resistance structure identified in the clustering analyses and suggest that resistance to one agent within this cluster may indicate a high probability of concurrent resistance to other agents in the same cluster. Nevertheless, treatment decisions should, whenever possible, be guided by isolate-level susceptibility testing together with the broader clinical context.

### 3.4. Comparison with Reports from Other Countries

Co-resistance patterns found in this study are not unique to Japan; similar patterns have been reported in other countries. In a report from the United States on antimicrobial drug resistance in *E. coli* from humans and food animals (1950–2002), concurrent resistance to tetracycline and streptomycin was the most common co-resistance phenotype (29.7%), followed by resistance to tetracycline and sulfonamide (29.0%); tetracycline, sulfonamide, and streptomycin (23.9%); tetracycline and ampicillin (18.8%); and tetracycline, ampicillin, streptomycin, and sulfonamide (12.9%) [27]. In Denmark, combined resistance to ampicillin, sulfamethoxazole, and tetracycline (ASuT) continued to be the most common multidrug-resistance profile among *E. coli* from pigs in 2024 [28].

### 3.5. MDR-Associated Resistance and Methodological Considerations

Our MDR analysis complements the unsupervised clustering findings by quantifying which individual drug resistances are associated with MDR (≥3 classes). Because the outcome (MDR ≥ 3 classes) is defined directly by combinations of the explanatory variables themselves, the purpose of this analysis was not to optimize prediction but to identify which drug resistances contribute most strongly to MDR classification. If feature importance merely reflected the outcome definition, importance rankings would be expected to remain largely invariant across ≥1-, ≥2-, and ≥3-class outcomes; however, we instead observed systematic shifts in importance, indicating that the model captures non-trivial structure beyond marginal resistance prevalence. Across all attribution measures (gain, permutation importance, SHAP), ampicillin consistently emerged as the dominant contributor in the context of MDR (≥3-class) classification. Therefore, using SHAP as one of the XAI approaches, we built models for 1-class, 2-class, and 3-class resistance outcomes to identify factors contributing to the prediction of co-resistance. For ≥1- and ≥2-class resistance, SHAP importance largely tracked resistance prevalence (tetracycline and streptomycin dominated). In contrast, the ≥3-class model shifted toward ampicillin, sulfamethoxazole–trimethoprim, and streptomycin, with tetracycline no longer in the top three—suggesting that tetracycline resistance alone may be widespread but not sufficiently specific for identifying the transition into MDR, whereas ampicillin resistance better captures membership in the more complex resistance modules that constitute ≥3-class MDR. This model-based finding—that ampicillin resistance was strongly associated with MDR—was consistent with the results of the MIC-correlation and Jaccard-similarity analyses. Because these approaches capture different aspects of the resistance structure, their convergence strengthens the robustness of our interpretation.

### 3.6. Limitations and Future Directions

Several limitations should be considered when interpreting these findings. First, our analysis is based on slaughterhouse sampling of healthy pigs and therefore reflects population-level outcomes rather than farm-level causal processes. Heterogeneity in farm management, regional antimicrobial use, and animal movement networks may contribute to the observed resistance patterns, but these factors cannot be resolved with the current sampling design. Second, breakpoint sources were not fully harmonized. Because CLSI does not provide a breakpoint for streptomycin of *E. coli*, we classified streptomycin resistance using the EUCAST cutoff value. Ideally, all breakpoints would be derived from a single guideline to maximize consistency. Third, the XGBoost/SHAP analyses identify features that are predictive of MDR classification, not causal drivers. Because SHAP-based feature importance quantifies contribution to the model’s predictions but does not differentiate between causality and correlation [29], it cannot by itself establish whether the observed associations are causal. Determining whether reductions in the use of specific antimicrobials can causally lower resistance requires study designs that directly evaluate changes in AMU and AMR within the same production system. In particular, farm-level intervention studies—where the usage of a particular drug or drug class is deliberately reduced and resistance outcomes are monitored longitudinally—are essential for establishing whether decreasing AMU leads to measurable declines in resistance. Fourth, the co-resistance patterns observed here are based only on samples from healthy pigs. Therefore, it is unclear whether these patterns are specific to pigs or also occur in other healthy food animals, such as cattle or poultry. Fifth, the feature-importance analysis may be influenced by how MDR was defined in this study. In the primary analysis, our MDR classification treated penicillins, first- and second-generation cephalosporins, and third- and fourth-generation cephalosporins as three separate categories, whereas some other drug groups were counted as a single category despite including multiple agents. Consequently, a single β-lactam resistance mechanism could contribute to resistance across multiple MDR categories. To examine the robustness of our findings, we repeated the feature-importance analysis using an alternative MDR definition in which all β-lactams were collapsed into a single category. The main pattern of feature importance was broadly preserved (Supplementary Figures S1 and S2), indicating that the importance of ampicillin resistance was not entirely driven by the β-lactam categorization adopted in the primary analysis. However, some dependence of feature-importance estimates on the MDR definition remains possible.

Future work should integrate genomic data to determine whether the ABPC/SM/TC and CP/ST resistance modules correspond to specific mobile genetic elements circulating in Japanese pig populations. Horizontal gene transfer mediated by plasmids, integrons, and other mobile elements is widely recognized as a central mechanism shaping AMR structure in livestock [30,31], and likely forms the genetic backbone of the co-resistance patterns observed in this study. In particular, a previous Japanese study using *E. coli* isolates from healthy pigs collected between 2004 and 2007 demonstrated, through conjugation experiments and whole-genome sequencing, that plasmids carrying combinations of resistance genes overlapping with the resistances highlighted here—including ampicillin, streptomycin, tetracycline, chloramphenicol, and trimethoprim resistance—could be transferred between bacteria [31]. This supports the plausibility that mobile genetic elements contribute substantially to the co-resistance structure identified in the present study. To clarify these mechanisms, we plan to perform whole-genome sequencing of *E. coli* isolates and investigate the underlying mobile genetic elements in detail.

## 4. Materials and Methods

### 4.1. Data Source and Isolate Selection

We used MIC data obtained annually from 2012 to 2023 for *E. coli* isolates from healthy pigs in Japan through JVARM surveillance. Sampling was conducted at slaughterhouses, where rectal fecal specimens were collected. Samples were inoculated onto deoxycholate hydrogen sulfide lactose (DHL) agar (Eiken Chemical Co., Ltd., Tokyo, Japan), and presumptive *E. coli* colonies were confirmed using Triple Sugar Iron (TSI) agar (Eiken Chemical Co., Ltd., Tokyo, Japan) and IMViC (Indole, Methyl Red, Voges–Proskauer and Citrate) tests using SIM medium and SC medium (Eiken Chemical Co., Ltd., Tokyo, Japan), MR-VP Broth (Nippon Becton Dickinson Company, Ltd., Tokyo, Japan), methyl red (Kanto Chemical Co., Inc., Tokyo, Japan), KOVACS indole reagent (Merck Ltd., Tokyo, Japan), and VP reagents A and B (Muto Pure Chemicals Co., Ltd., Tokyo, Japan). The final dataset comprised 1312 isolates collected over 12 years (2012–2023), with annual sample sizes as follows: 2012 (193), 2013 (126), 2014 (87), 2015 (96), 2016 (89), 2017 (83), 2018 (83), 2019 (80), 2020 (93), 2021 (102), 2022 (136), and 2023 (144). For geographical characterization, information on the prefecture of the source farm for each sampled animal was recorded when available. Japan is divided into 47 prefectures, and the numbers of prefectures represented in the dataset were 25 in 2013, 23 in 2014, 23 in 2015, 20 in 2016, 25 in 2017, 25 in 2018, 27 in 2019, 30 in 2020, 28 in 2021, 31 in 2022, and 36 in 2023. For isolates collected in 2012, information on the prefecture of the source farm was unavailable. Instead, samples were obtained at slaughterhouses in Tokyo, Osaka, and Kagoshima. These slaughterhouses served farms from multiple prefectures and were located in geographically distinct regions of Japan.

### 4.2. MIC Values and Interpretation

MICs were determined using the broth microdilution method in accordance with Clinical and Laboratory Standards Institute (CLSI) recommendations [32]. Twelve antimicrobials routinely tested in JVARM for this population were included in this study: ampicillin (ABPC), cefazolin (CEZ), cefotaxime (CTX), chloramphenicol (CP), ciprofloxacin (CPFX), colistin (CL), gentamicin (GM), kanamycin (KM), nalidixic acid (NA), streptomycin (SM), sulfamethoxazole–trimethoprim (ST), and tetracycline (TC). Clinical breakpoints were derived from CLSI M100 [33], except for streptomycin. The resistance thresholds (μg/mL) used were: ABPC 32, CEZ 8, CL 4, CP 32, CPFX 1, CTX 4, GM 8, KM 64, NA 32, ST 76/4, and TC 16. Isolates with MIC values greater than or equal to the corresponding breakpoint were classified as resistant. Because a CLSI breakpoint for streptomycin in *E. coli* is not available, resistance to streptomycin was classified using the European Committee on Antimicrobial Susceptibility Testing (EUCAST) epidemiological cut-off value (ECOFF) [34]. Since the ECOFF for streptomycin in *E. coli* is 16 µg/mL, strains with MIC values of 32 µg/mL or higher were classified as resistant, as ECOFF represents the upper limit of the MIC distribution for wild-type populations.

### 4.3. Temporal Trend Analysis

Data preprocessing and wrangling were performed in Python (version 3.11.5) using pandas (version 2.2.3) [35]. Temporal trends in annual resistance prevalence were evaluated using the Mann–Kendall trend test, and the magnitude of monotonic change was summarized using Sen’s slope. These trend analyses were conducted in R (version 4.3.0) with the package trend (version 1.1.6) [36]. Figures were generated using plotly (version 5.18.0) [37].

### 4.4. MIC-Profile Similarity and Hierarchical Clustering

MIC values were scaled to a 0–1 range within each antimicrobial agent using MinMaxScaler (scikit-learn version 1.6.1) [38] to facilitate cross-drug comparison of MIC patterns. This preprocessing allows correlation to reflect co-variation in isolate-level MIC patterns (relative increases/decreases) across drugs, rather than being dominated by drug-specific MIC ranges or dilution series, which are not directly comparable between antimicrobials.

Drug–drug similarity was quantified using pairwise Pearson correlation coefficients computed from the normalized MIC profiles across isolates. A correlation-based distance matrix was then defined as 1 − r, where r is the correlation coefficient. Hierarchical clustering of antimicrobials was performed using average linkage on this distance matrix. In parallel, hierarchical clustering of isolates (samples) was performed using Euclidean distance with Ward linkage. Clustered heatmaps were generated to visualize the resulting structures. Distance computation and hierarchical clustering were implemented using SciPy (version 1.16.3) [39], and visualization was performed using seaborn (version 0.13.2) [40].

Pairwise correlation coefficients were summarized for all antimicrobial pairs (66 pairs). In addition, to identify a centrally positioned antimicrobial in correlation space, a medoid was defined as the drug minimizing the sum of correlation distances to all other drugs, i.e., minimizing ∑(1 − r).

### 4.5. Similarity of Binary Resistance Patterns and Clustering

To characterize co-occurrence of resistance across antimicrobials, MIC results were first converted to binary resistance indicators (0 = susceptible, 1 = resistant) based on the thresholds described above. Pairwise Jaccard similarity was then computed between antimicrobials, and a Jaccard distance matrix was defined as 1 − Jaccard similarity.

Hierarchical clustering was performed using average linkage on the Jaccard distance matrix and visualized as a clustered heatmap using a binary colormap (susceptible vs. resistant). Distance computation and clustering were conducted using SciPy (version 1.16.3) [39], and figures were produced using seaborn (version 0.13.2) [40]. The medoid in Jaccard space was defined as the antimicrobial minimizing the sum of Jaccard distances to all other drugs.

In addition, to assess whether the observed pairwise Jaccard similarities exceeded those expected by chance given the marginal resistance frequencies of each drug, we performed a Monte Carlo simulation under an independence-based null model. For each antimicrobial pair, resistance status was simulated independently for each isolate using Bernoulli draws with probabilities set to the observed resistance proportions of the respective drugs, while keeping the total number of isolates equal to that in the original dataset (1312). A total of 10,000 Monte Carlo replicates were generated for each pair, and the Jaccard similarity coefficient was calculated for each simulation to obtain a null distribution under independence. The null distribution was summarized by its median and central 95% interval (2.5th–97.5th percentiles). The extremeness of the observed Jaccard coefficient was quantified using a one-sided empirical *p* value (upper tail), defined as the proportion of simulated Jaccard coefficients greater than or equal to the observed value, computed using the standard Monte Carlo adjustment (k + 1)/(N + 1) to avoid zero *p* values. To control the family-wise error rate across all antimicrobial pairs, these *p* values were adjusted using the Bonferroni method. Antimicrobial pairs were considered to show statistically significant co-resistance when the Bonferroni-adjusted *p* value was less than 0.05. In the Monte Carlo simulation, Jaccard similarity coefficients were calculated using scikit-learn (version 1.6.1) [38]. Binomial (Bernoulli) simulations were performed using NumPy (version 2.3.5) [41] with a fixed random seed (seed = 42), and multiple-testing correction was applied using statsmodels (version 0.14.6) [42]. Data visualization was conducted using matplotlib (version 3.10.0) [43].

### 4.6. Multidrug Resistance (MDR) Definition

The MDR definition was based on the framework proposed by Magiorakos et al. [23], with adaptations to match the antimicrobial panel available in JVARM. Antimicrobials were grouped into the following classes, and class-level resistance was defined as resistance to at least one agent within the class:

- Aminoglycosides: GM, KM, SM.
- 1st and 2nd generation cephalosporins: CEZ.
- 3rd and 4th generation cephalosporins: CTX.
- Quinolones/fluoroquinolones: NA, CPFX.
- Folate pathway inhibitors: ST.
- Penicillins: ABPC.
- Phenicols: CP.
- Polymyxins: CL.
- Tetracyclines: TC.

For each isolate, the number of resistant classes was counted, and isolates resistant to ≥3 classes were classified as MDR-positive.

### 4.7. Model-Based Estimation of Feature Importance for MDR

To identify drug-level resistance features most strongly associated with MDR classification, an XGBoost classifier (xgboost version 2.1.4) [20] was trained using binary resistance indicators (0/1) for each antimicrobial as explanatory variables and MDR status (≥3 classes) as the outcome. The model was trained using default hyperparameters.

To obtain robust estimates of feature importance, we fitted XGBoost models under repeated stratified cross-validation (5 folds × 10 repeats), yielding 50 trained models; feature importance was quantified across these models using three complementary approaches:

- Gain-based feature importance from XGBoost, derived from the trained model and reflecting split improvements on the training data.
- Permutation feature importance (PFI), defined as the decrease in balanced accuracy on the test data after permuting each feature.
- SHAP values computed using TreeExplainer (shap version 0.50.0) [44], calculated on the test data and summarized as mean absolute SHAP values across test samples.

For all three feature-importance measures, feature scores were aggregated across cross-validation folds using the median. Balanced accuracy was used as the primary performance metric because the MDR outcome was moderately imbalanced (MDR prevalence: 40%). Balanced accuracy and permutation importance were computed using scikit-learn (version 1.6.1) [38]. To ensure reproducibility, a fixed random seed (seed = 42) was applied to all stochastic processes, including data splitting, model initialization, and permutation-based feature importance estimation. Visualization was performed with seaborn (version 0.13.2) [40].

### 4.8. Feature Importance Analysis Using Alternative MDR Outcome Definitions (≥1, ≥2, and ≥3 Resistant Classes)

To examine how feature attributions change with the definition of co-resistance, we defined outcomes based on resistance to ≥1, ≥2, or ≥3 antimicrobial classes using the same class-count rule. We then conducted separate XGBoost/SHAP analyses for each outcome using the same repeated cross-validation design described above. Software and library versions were identical to those used above.

## 5. Conclusions

Using 12 years of JVARM data from healthy pigs (2012–2023), we characterized both the prevalence of resistance and the co-occurrence structure of resistance across antimicrobials. Resistance remained highest for tetracycline, with moderate levels for streptomycin, ampicillin, sulfamethoxazole–trimethoprim, and chloramphenicol, whereas cefotaxime, ciprofloxacin, and colistin stayed at low prevalence. Correlation- and Jaccard-based clustering indicated non-random structure in resistance, highlighting two closely positioned co-resistance groupings in the clustering space: ABPC/SM/TC and CP/ST. Ampicillin was identified as the medoid in both MIC-based and resistance-profile similarity spaces, with streptomycin also positioned near the center in both structures. In the MDR analysis (≥3 classes), under the outcome definition used in this study, ampicillin resistance emerged as a leading resistance feature associated with MDR across feature-importance metrics, with its contribution increasing as the outcome definition shifted from ≥1- or ≥2-class resistance to ≥3-class resistance. These patterns imply that reducing resistance in the co-resistance groupings may require coordinated stewardship across linked classes, while more independent resistances may respond to class-specific interventions. As our machine-learning analyses quantify predictive associations rather than causality, targeted intervention and genomic studies are needed to confirm mechanisms and estimate the impact of AMU changes.

## Data Availability

The data supporting the findings of this study are provided as Supplementary Materials.

## Supplementary Materials

The following supporting information can be downloaded at: https://www.mdpi.com/article/10.3390/antibiotics15050441/s1, Table S1: Supplemental Data—MIC data. Figure S1: Sensitivity analysis of feature importance for MDR prediction across repeated cross-validation under an alternative MDR definition in which all β-lactams were grouped into a single category. Figure S2: Sensitivity analysis of drug-level feature importance across ≥1-class, ≥2-class, and ≥3-class resistance outcomes under an alternative MDR definition in which all β-lactams were grouped into a single category.
