# Supplementary material for: Co-Resistance Structure and Multidrug Resistance-Associated Antimicrobials in Escherichia coli from Healthy Pigs in Japan: A Computational Analysis of JVARM Data, 2012–2023
Source: Antibiotics (Basel). 2026 Apr 29;15(5):441. doi: 10.3390/antibiotics15050441 (PMC13203292; doi:10.3390/antibiotics15050441)
Supplement: Supplementary file 1 [file antibiotics-15-00441-s001.zip › antibiotics-4274104-Supplementary figures.pdf]

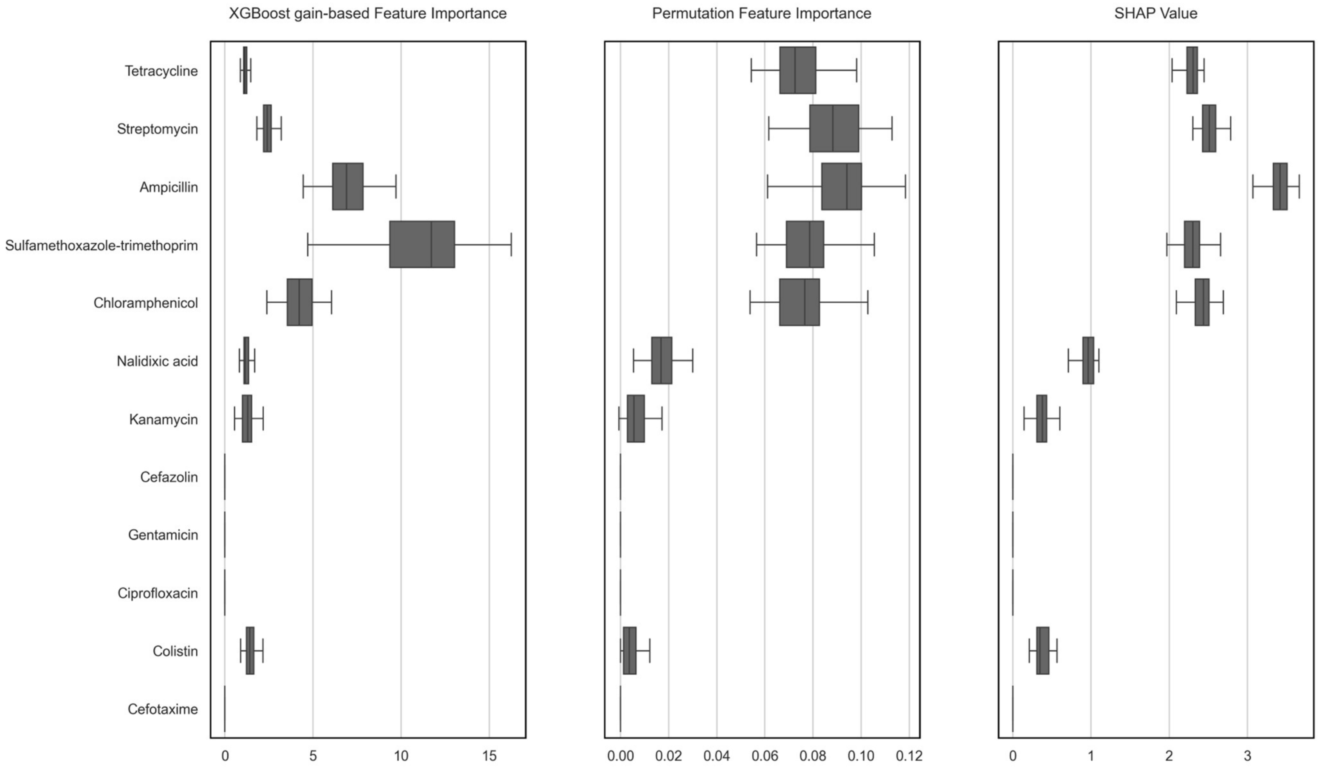

Figure S1. Sensitivity analysis of feature importance for MDR prediction across repeated cross-validation under an alternative MDR definition in which all β-lactams were grouped into a single category.

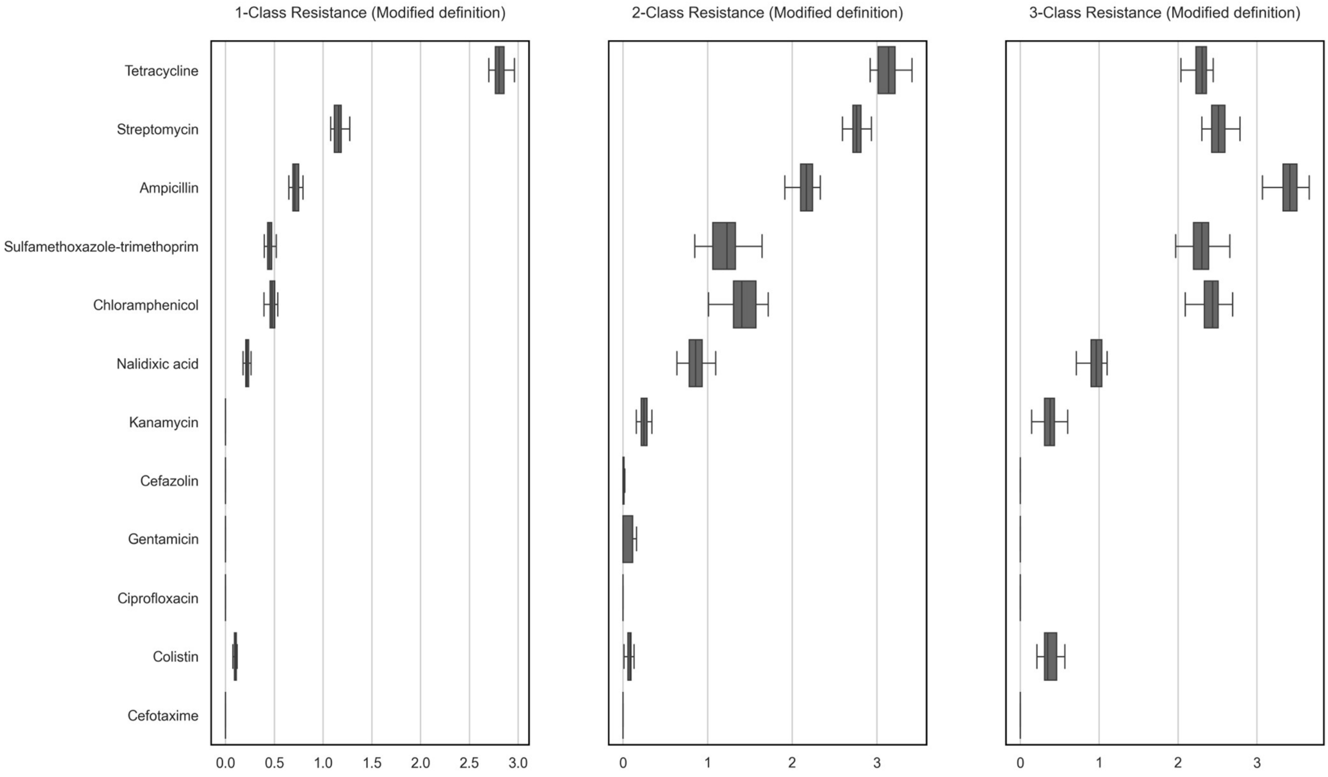

Figure S2. Sensitivity analysis of drug-level feature importance across ≥1-class, ≥2-class, and ≥3-class resistance outcomes under an alternative MDR definition in which all β-lactams were grouped into a single category.
